# Supplementary material for: Downregulation of hepatic lncRNA Gm19619 improves gluconeogenesis and lipogenesis following vertical sleeve gastrectomy in mice
Source: Commun Biol. 2023 Jan 27;6:105. doi: 10.1038/s42003-023-04483-0 (PMC9883214; doi:10.1038/s42003-023-04483-0)
Supplement: Supplementary file 2 — Supplementary Information [file 42003_2023_4483_MOESM2_ESM.pdf]

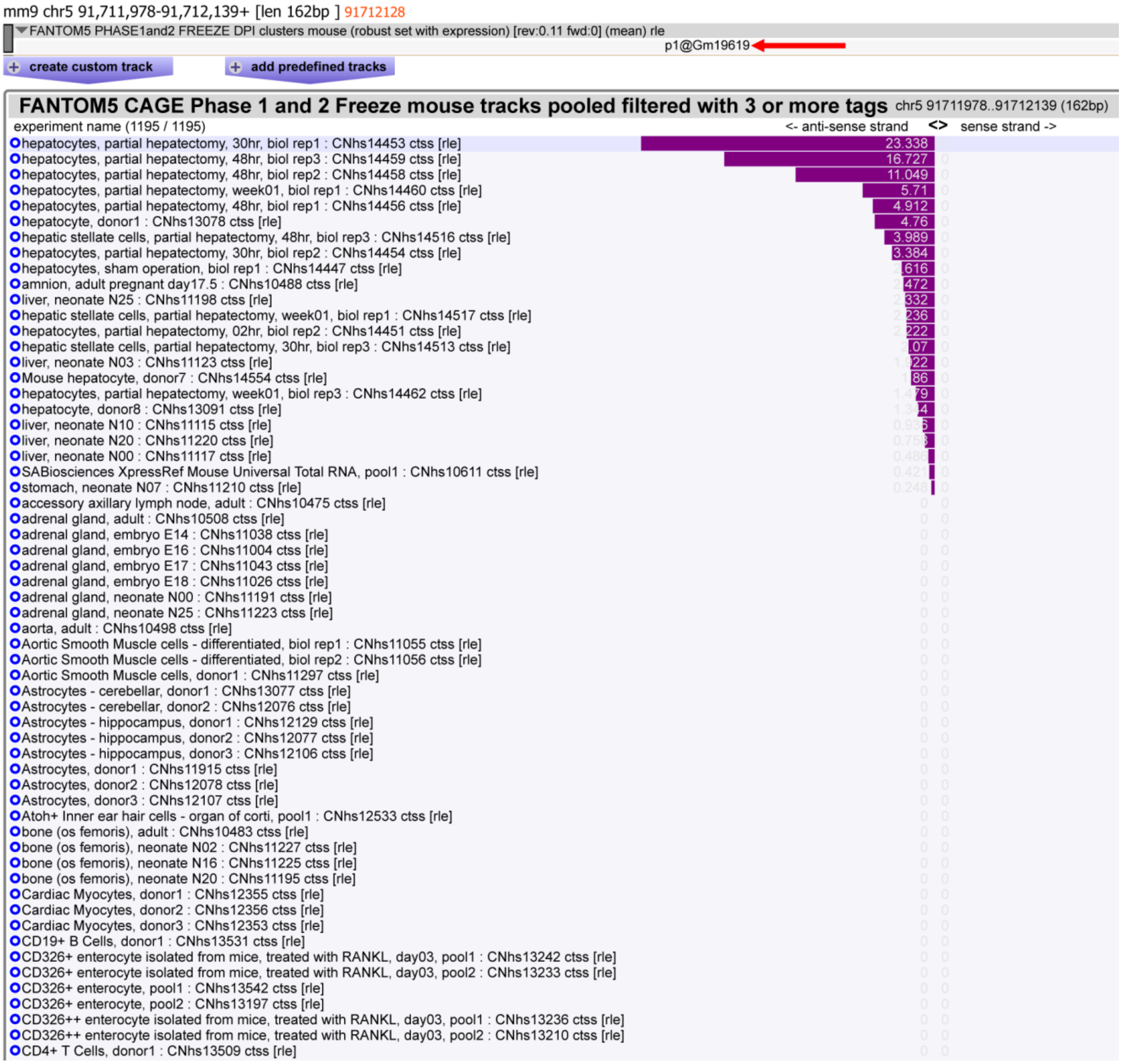

Supplementary Figure 1. The sorted data in Fantom5 database indicates that *Gm19619* is only transcribed in the murine liver, but not other tissues.

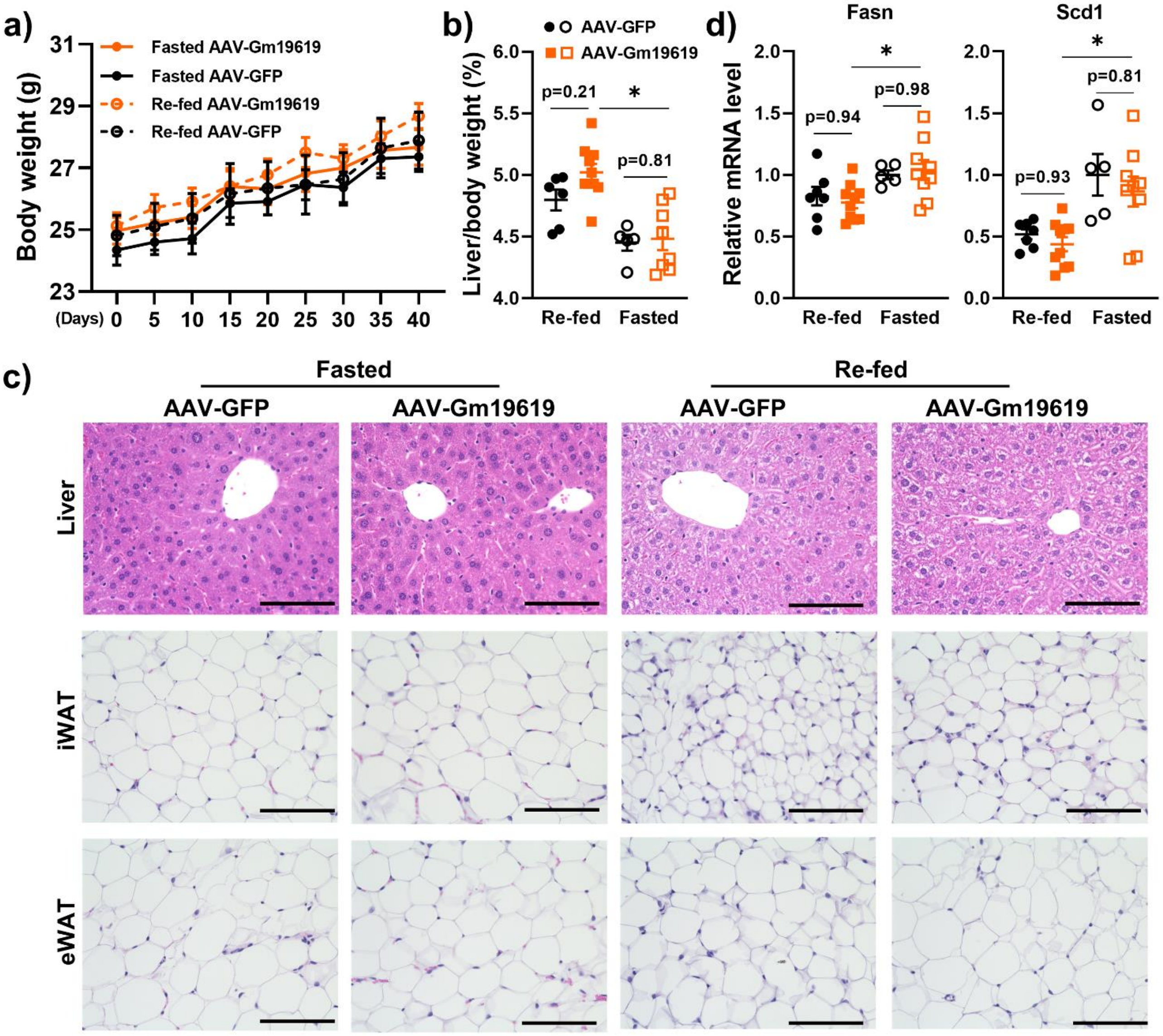

Supplementary Figure 2. Forced transcription of lncRNA *Gm19619* did not affect the body weight and lipid metabolism in chow-fed mice. (**a-b**) Body weight (**a**) and liver/body weight ratio (**b**) of mice from fasted (n=7) and re-fed (n=7) AAV-GFP, fasted (n=9) and re-fed (n=11) AAV-Gm19619 after AAV injection. (**c**) Representative hematoxylin and eosin staining images of the liver, iWAT and eWAT sections. Scale bar, 100 μm. (**d**). Relative mRNA levels of *Fasn* and *Scd1* (n=5-10). Two-tailed unpaired t-test. Error bars represent the SEM. * P < 0.05.

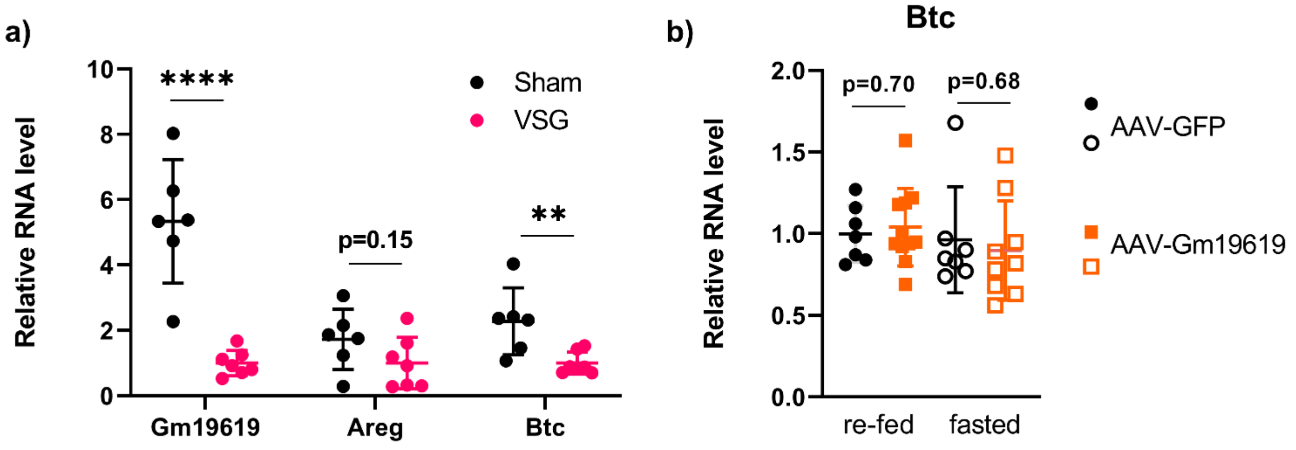

Supplementary Figure 3. Gm19619 did not regulate the transcription of its nearby genes. (**a**) The relative RNA levels of *Gm19619, Areg,* and *Btc* in Sham (n=6) and VSG (n=7) mice liver. (**b**) The relative mRNA levels of *Btc* in *Gm19619* forced transcribed and control mice (n=7-11). Two-tailed unpaired t-test. Error bars represent the SEM. ** P < 0.01, **** P < 0.0001.

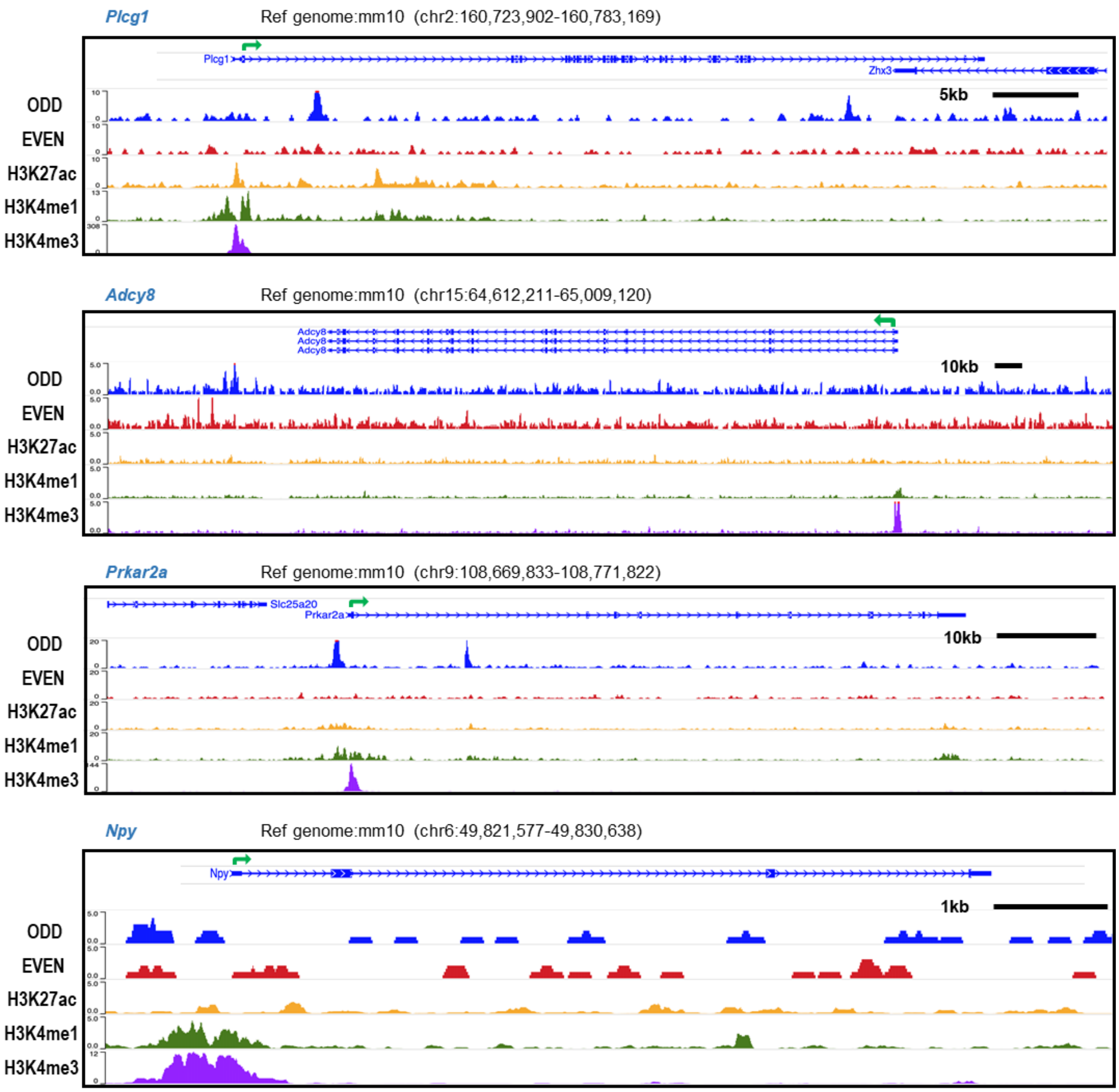

Supplementary Figure 4. ChIRP-seq signals indicated that *Gm19619* bound to the gene locus of several leptin receptor pathway genes: *Plcg1*, *Adcy8*, *Prkar2a*, and *Npy*. H3K27ac, H3K4me1, and H3K4me3 signals from ENCODE mouse liver ChIP-seq data were shown as controls. mm10 was shown as the reference genome.

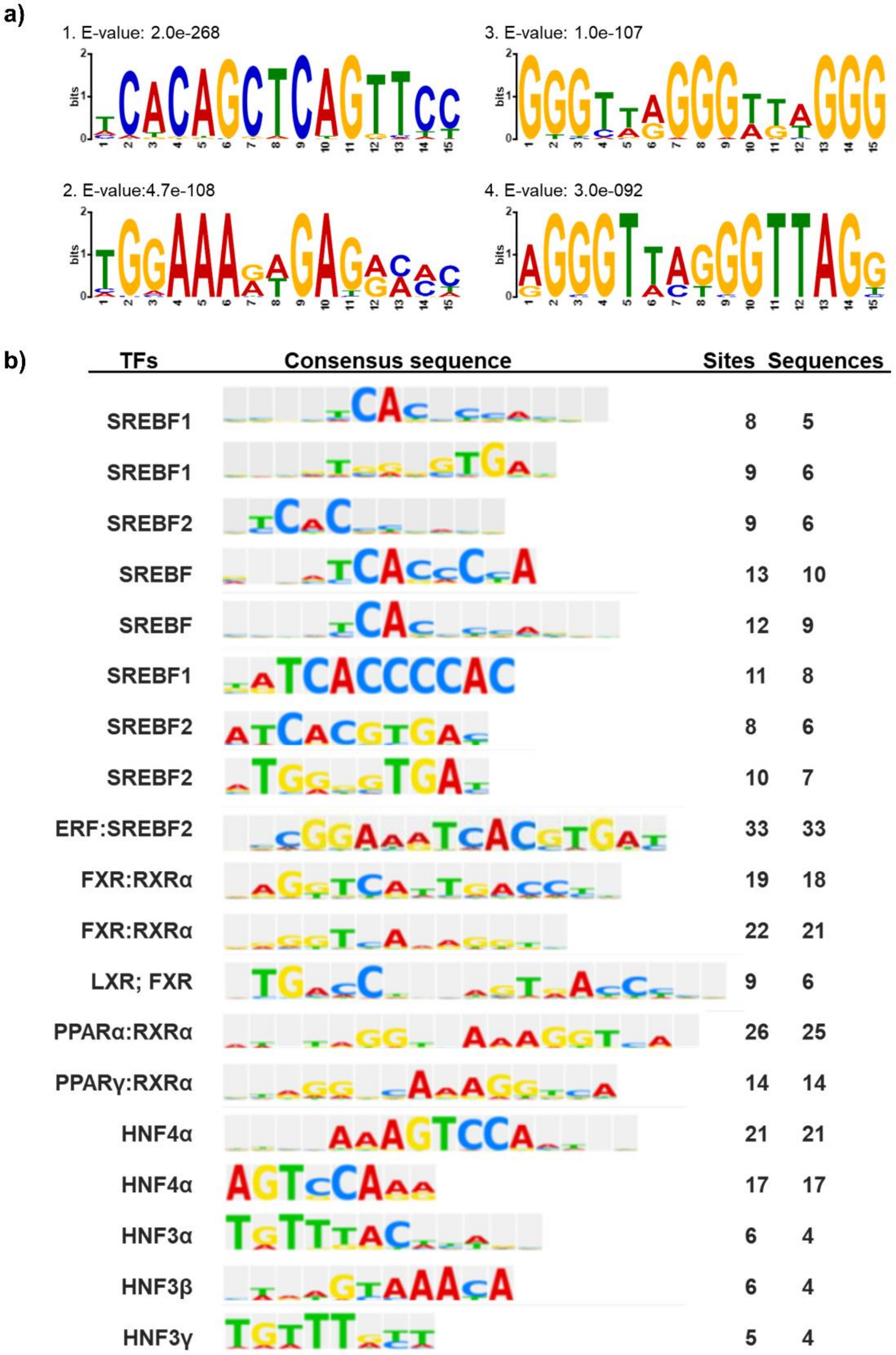

Supplementary Figure 5. The overlapping peak sequences were used to run TRANSFAC2.0 to find transcription factors. Several transcription factors, including SREBF1, SREBF2, and HNF4α, were identified by TRABSFAC2.0.

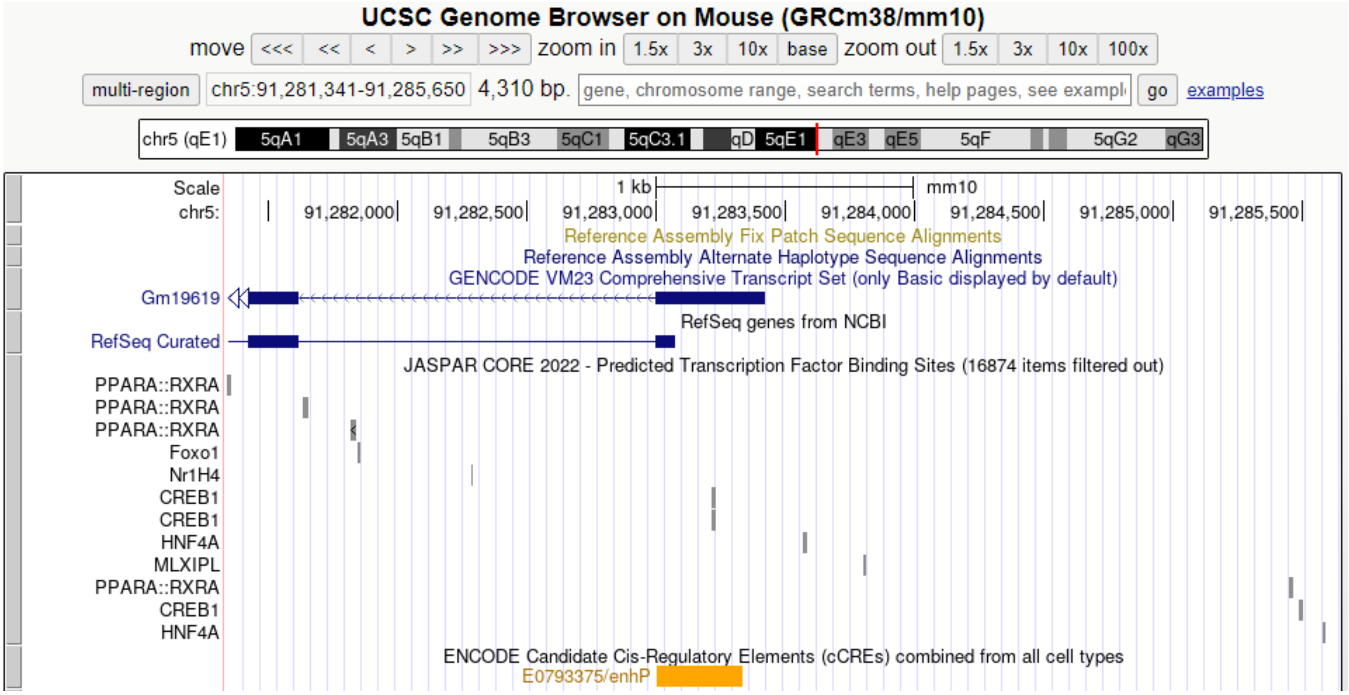

Supplementary Figure 6. The prediction of potential transcription factors binding around Gm19619 promoter region by JASPAR. Note that the putative promoter region of Gm19619 may be bound by CREB1, PPARα, FXR, or other TFs known to regulate glucose metabolism in fasted and fed states.

Fig. 2c

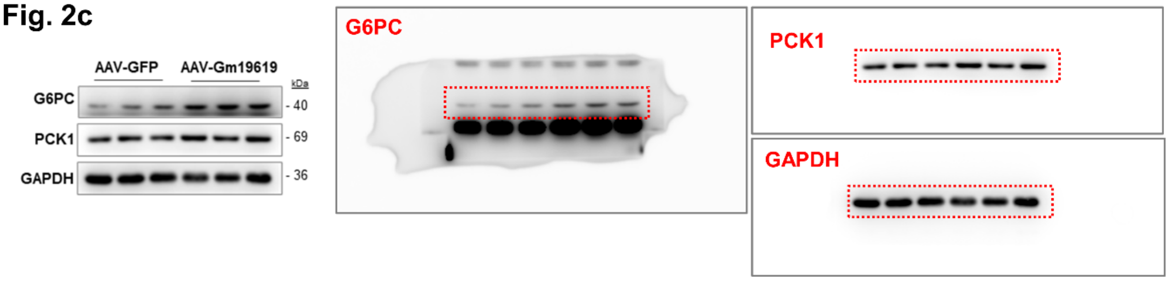

Fig. 3d

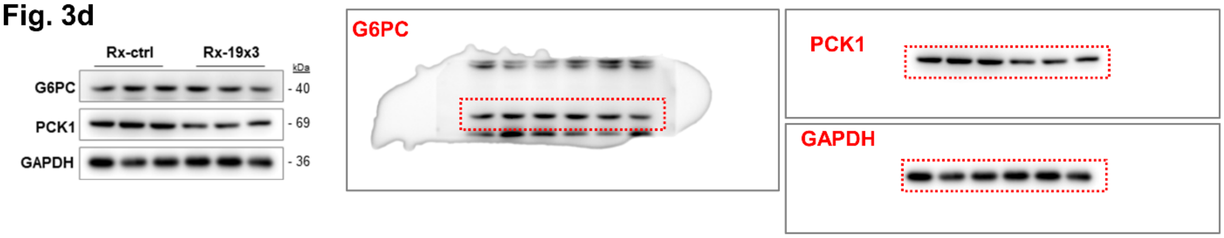

Fig. 5h

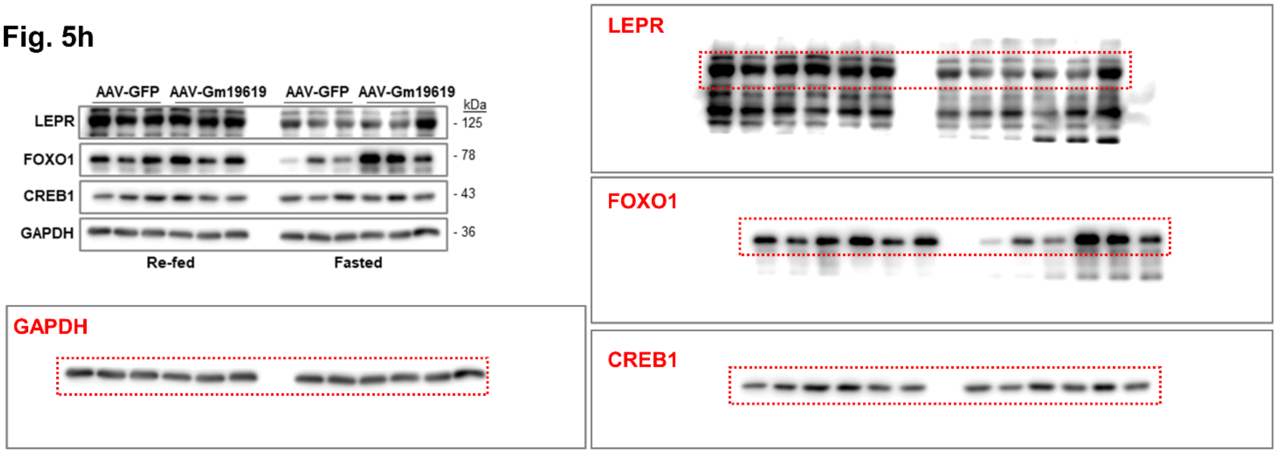

Fig. 5j

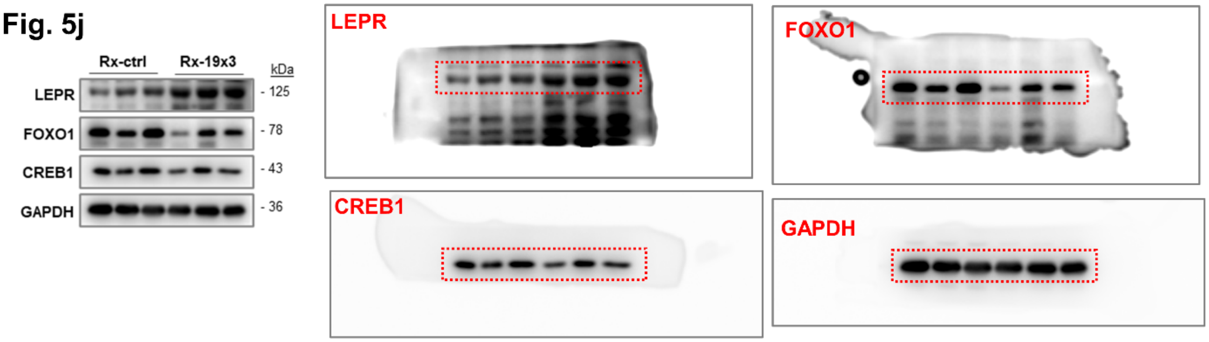

Supplementary Figure 7. Uncut western blot images.

Supplementary Table 1. Sequences of *Gm19619* ChIRP probes.

Name	Sequence
Gm19619_probe_1	aaggagagtgaagtggcagt
Gm19619_probe_2	gacactgagcccaatagaat
Gm19619_probe_3	tgtgccatgtgaaagcagag
Gm19619_probe_4	ttcaaggtcattgacacacc
Gm19619_probe_5	gaaaagacccggacttggta
Gm19619_probe_6	actactgctacatgagatct
Gm19619_probe_7	gggaactgagctgtgagatg
Gm19619_probe_8	cagattgtggatagtgatga
Gm19619_probe_9	ctatgggttctggacagctc
Gm19619_probe_10	aagcacatttccgaaggagt
Gm19619_probe_11	tatctttgagcttcgagacc
Gm19619_probe_12	tcacccagatgagctaacaa
Gm19619_probe_13	cagatgatacgatgtcccaa
Gm19619_probe_14	gtggcatactgtcatatata

Supplementary Table 2. Sequences of primers for QPCR.

Gene Name	Forward Primer (5'-3')	Reverse Primer (5'-3')
Gm19619	AAGCATCAGAGTCCAGGGTC	CGGAAATGTGCTTGCCCTAA
Rnu2	GGAGTTGGAATAGGAGCTTGC	TGCACCGTTCCTGGAGGTAC
Gapdh	AGGTCGGTGTGAACGGATTTG	TGTAGACCATGTAGTTGAGGTCA
Rplp0	TCGGGTCCTAGACCAGTGTTC	AGATTCGGGATATGCTGTTGGC
G6pc	CGACTCGCTATCTCCAAGTGA	GTTGAACCAGTCTCCGACCA
Pck1	CTGCATAACGGTCTGGACTTC	CAGCAACTGCCCGTACTCC
Srebf1	TGACCCGGCTATTCCGTGA	CTGGGCTGAGCAATACAGTTC
Scd1	TTCTTGCGATACACTCTGGTGC	CGGGATTGAATGTTCTTGTCGT
Fasn	GGAGGTGGTGATAGCCGGTAT	TGGGTAATCCATAGAGCCCAG
Cd36	ATGGGCTGTGATCGGAACTG	GTCTTCCCAATAAGCATGTCTCC
Lepr	TGGTCCCAGCAGCTATGGT	ACCCAGAGAAGTTAGCACTGT
Plcg1	ATCCAGCAGTCCTAGAGCCTG	GGATGGCGATCTGACAAGC
Prkar2a	GAGGAGGATAACGATCCAAGGG	TGCTCGTCAGTTTTGACAATCTT
Areg	TGCACAGTCCCGTTTTCTTG	CAGTGCACCTTTGGAAACGA
Btc	TTCTCACAGATGCAGGAGGG	GGGAACACAACCAGAACACC
Acsl1	TGCCAGAGCTGATTGACATTC	GGCATACCAGAAGGTGGTGAG
Pnpla2	CAACGCCACTCACATCTACGG	GGACACCTCAATAATGTTGGCAC
Lipe	CCAGCCTGAGGGCTTACTG	CTCCATTGACTGTGACATCTCG
Cpt1a	CTCCGCCTGAGCCATGAAG	CACCAGTGATGATGCCATTCT
Cpt1b	GCACACCAGGCAGTAGCTTT	CAGGAGTTGATTCCAGACAGGTA
